# Gamma‐secretase modulation: from bench to clinic

**DOI:** 10.1002/alz.090510

**Published:** 2025-01-09

**Authors:** Irene Gerlach, R. Tortelli, T. Mueggler, L. Müller, L. Lindemann, R. Croney, P. Piosik, S. DeBuck, T. Vardar5, K. Baumann

**Affiliations:** ^1^ Roche, Basel, na Switzerland

## Abstract

Amyloid β (Aβ) has been confirmed as a therapeutic target in AD by recent findings in Phase 3 trials with anti‐Aβ antibodies. Modulators of γ‐secretase (GSMs) are an emerging complementary approach to target amyloid. GSMs “modulate” the interaction between γ‐secretase and amyloid precursor protein (APP), leading to a reduced production of long, amyloidogenic Aβ42 and Aβ40 and to concomitantly increased levels of the shorter, non amyloidogenic Aβ37 and Aβ38. This change in processivity of γ‐secretase results in a reduced formation of soluble and insoluble Aβ aggregates and plaque deposits. Unlike inhibitors of γ‐secretase or BACE1, GSMs do not inhibit the activity of γ‐secretase and thus do not block the processing of APP and of other substrates. In addition, non‐clinical studies showed that GSMs may have beneficial effects on synaptic integrity and Aβ‐associated neuro‐inflammation.

The clinical relevance of higher Aβ37 and Aβ38 ‐levels for a reduced risk of cognitive decline have been described in observational cohorts (vonKienlin et al. 2018; Cullen et al. 2022). Nevertheless, to date the effects of GSMs on brain function and course of disease in people living with AD have not been investigated. Our novel orally available GSM RG6289 has completed the Phase 1 study in healthy young and elderly volunteers. Safety, pharmacokinetic and pharmacodynamic data from this study support the further clinical development of the molecule. The Phase 2 study in individuals with AD is expected to start this year. Challenges of developing in clinic a new mechanism of action, as well as considerations regarding how to best position this molecule for AD will be discussed in this presentation.

